# Fast Classification of Meat Spoilage Markers Using Nanostructured ZnO Thin Films and Unsupervised Feature Learning

**DOI:** 10.3390/sl30201578

**Published:** 2013-01-25

**Authors:** Martin Längkvist, Silvia Coradeschi, Amy Loutfi, John Bosco Balaguru Rayappan

**Affiliations:** 1 Center for Applied Autonomous Sensor Systems, Örebro University, SE-701-82, Örebro, Sweden; E-Mails: marting.langkivst@oru.se (M.L.); silvia.coradeschi@oru.se (S.C.); amy.loutfi@oru.se (A.L.); 2 Centre for Nanotechnology & Advanced Biomaterials (CeNTAB) & School of Electrical & Electronics Engineering, SASTRA University, Thanjavur 613 401, Tamil Nadu, India; E-Mail: rjbosco@ece.sastra.edu

**Keywords:** electronic nose, sensor material, representational learning, fast multi-label classification

## Abstract

This paper investigates a rapid and accurate detection system for spoilage in meat. We use unsupervised feature learning techniques (stacked restricted Boltzmann machines and auto-encoders) that consider only the transient response from undoped zinc oxide, manganese-doped zinc oxide, and fluorine-doped zinc oxide in order to classify three categories: the type of thin film that is used, the type of gas, and the approximate ppm-level of the gas. These models mainly offer the advantage that features are learned from data instead of being hand-designed. We compare our results to a feature-based approach using samples with various ppm level of ethanol and trimethylamine (TMA) that are good markers for meat spoilage. The result is that deep networks give a better and faster classification than the feature-based approach, and we thus conclude that the fine-tuning of our deep models are more efficient for this kind of multi-label classification task.

## 1. Introduction

Nanostructured zinc oxide (ZnO) thin films are showing an increasing potential as sensing components in electronic nose instruments. As described in [1-3], these materials have been successfully applied in the detections of volatile organic compounds particularly associated to markers of meat spoilage. With certain markers such as ethanol, the nanostructured ZnO thin films have shown detection levels in the ppb levels, thus outperforming traditional metal oxide semiconductors based on SnO_2_. However, as illustrated in [3], a potential drawback is the longer settling time required, and therefore traditional methods that rely on sensors reaching a steady state are less suitable for applications requiring a fast response. In this paper, we propose to circumvent this shortcoming via a fast classification algorithm that does not require that the sensors reach a steady state, but instead uses transient information from the response characteristic of the sensor when exposed to an analyte. The application area that we are considering is food safety and in particular we aim at developing an instrument that can be used *in situ* for rapid identification of meat spoilage [4–8].

In the past two decades, the awareness about food safety, particularly with respect to specific pathogenic bacteria, has increased. This is especially true in the case of meat and fish, where microbial spoilage can be dangerous for humans, and where there is a clear requirement for a rapid and accurate detection system [9–11]. Traditionally, fish and meat quality is assessed by examining the structure of the food (texture, tenderness, flavor, juiciness, color), or by detecting the microorganism and its count, or by detecting the gases generated by these microorganisms. A number of techniques have been used to examine the quality of the meat, namely instrumental mechanical methods [12,13], the ultrasound technique [14,15], as well as optical spectroscopy [16,17], microscopy [18,19] and magnetic resonance [20,21] methods. These techniques have several disadvantages: they are destructive of the sample, they require complex sample preparation and data analysis, and they can be quite costly. Using electronic nose technologies, that is, an array of partially selective gas sensors together with pattern recognition techniques, gives a rapid quality discrimination with great accuracy and efficiency [22–26]. For applications regarding meat spoilage, two typical markers of interest are ethanol and trimethylamine [27–31].

Discrimination of the transient response has shown to be successful in previous investigations with SnO_2_ semiconductors [32]. The works considering the transient response can be divided between those using solely transient response, and those which use both features extracted from the transient, as well as the steady-state phase. In the latter case, the features used include modeling the signal with a multi-exponential function, features extracted using polynomial and exponential functions, ARX models, wavelets, and simple heuristics. In these works, classification performance improves when taking into account both the transient and the steady-state properties of the signal response. Works which only use the transient response have been mainly applied to mobile robotic olfaction where so-called open sampling systems are used. These systems have an open exposed sensor array interacting directly with the environment while the robot is moving [33–35]. In these works, only the transient information is available, and successful classification has been obtained using various feature extraction techniques (Fast Fourier transform, Discrete Wavelet Transform) together with a support vector machine classifier [35,36].

In this paper, we investigate the use of transient analysis on nanostructured zinc oxide thin films. Furthermore, in order to circumvent the tendency to rely on handmade features when extracting relevant data from the signal response, this paper investigates the use of unsupervised feature learning, where, namely, deep networks that include stacks of restricted Boltzmann machines and stacks of auto-encoders are applied. Previously, it has been demonstrated that a stack of restricted Boltzmann machines can be used for discrimination of a typical three phase signal (baseline, exposure and recovery) from commercially available tin dioxide semiconductor sensors [37]. However, this paper investigates the possibility to apply unsupervised feature learning considering only information from the transient response collected from nanostructured ZnO thin films both undoped and doped with Mn and F. Our results show that it is possible to improve classification speed from 14 min (in some cases) to less than 30 s.

## 2. Materials & Methods

### 2.1. Materials

Nanostructured undoped ZnO, Mn-doped ZnO, and F-doped ZnO thin films were deposited using the spray pyrolysis technique over the surface of ultrasonically cleaned glass substrates at optimized deposition conditions. The structural, morphological, optical, electrical properties, and the sensing characteristics (transient response, response and recover times) towards few ppm levels of ethanol and trimethylamine of these films are reported in [1–3]. From the investigation on the influence of precursor concentrations on the structural, morphological, optical, and electrical properties of ZnO thin films deposited with the 0.05 M of zinc acetate dihydrate 0.004 M of manganese acetate in 0.05 M of zinc acetate dihydrate, 0.002 M of ammonium fluoride in 0.05 M of zinc acetate dihydrate and 0.06 M of cadmium acetate dihydrate in 0.04 M of zinc acetate dihydrate as precursor concentrations were taken into consideration for sensing studies. In this investigation, these developed sensing elements have been used to collect sensing data for various concentrations of ethanol and trimethylamine (TMA) at the optimized operating temperature using the methodology reported in [1–3].

Mn-doped ZnO is an n-type semiconducting material. When it is exposed to the atmosphere, the oxygen molecules react with its surface and capture electrons from its conduction band. This in turn leads to a decrease in the electron concentration and, hence, increases the surface resistance until equilibrium. The stabilized surface resistance forms the baseline for the sensing studies. When the reducing vapours like ethanol or TMA are presented to the sensing element, the vapour reacts with surface-adsorbed oxygen species and increases the electrons concentration on the surface. As a result, the surface resistance decreases from the stabilized baseline and attains saturation. This change in surface resistance has a strong correlation with the concentration of ethanol/TMA in dry air atmospheric conditions [3].

**Figure 1.**
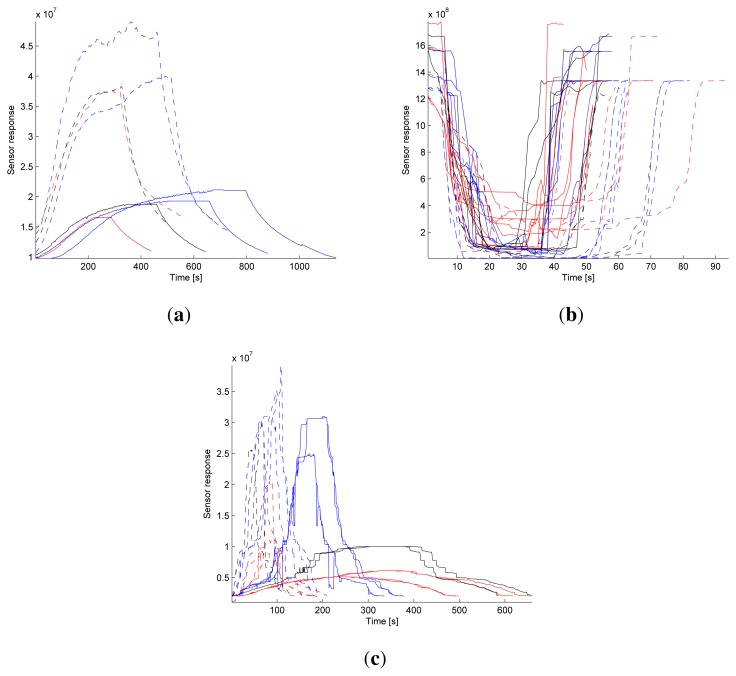
Sensor response for (**a**) undoped ZnO, (**b**) Mn doped ZnO, and (**c**) F doped ZnO. Solid line is the response towards ethanol and dotted line is the response towards trimethylamine. The color indicates ppm level where red, black, and blue represent low, medium, and high ppm level, respectively. Best viewed in color.

In the case of F-doped ZnO, the baseline formation is very similar to the Mn-doped ZnO case. However, the response towards ethanol and TMA is the opposite of the undoped ZnO and Mn-doped ZnO sensing behavior, see Figure 1. This is because of the high electronegative fluorine sites restrict the flow of electrons injected by the reducing nature of ethanol/TMA which in turn enhanced the scattering of the electrons at the grain boundaries. As a result, the surface resistance increases form the baseline and attains saturation [1].

### 2.2. Sample Collection

Spray-deposited nanostructured thin films with the dimension of 1 cm × 1 cm were used as the sensing element. Electrical contacts were made using copper wire and silver paste on the film to obtain Ohmic contact. The response of the selected films was observed at an optimized operating temperature using a homemade volatile organic compound (VOC) testing chamber of 5 L capacity with a digital thermostat coupled with a compact heater and a septum provision to inject desired concentration of VOCs using a micro-syringe. Changes in the electrical resistance of the films were recorded using an electrometer (Model 6517A, Keithley, Germany) as a function of time during the process of injection and venting.

As soon as the resistance was stabilized in dry air atmosphere, the baseline was fixed. Then the desired concentration of the target gas was injected into the glass chamber using a micro-syringe. Once the change in resistance became stable or saturated in the presence of target gas, the target gas was evacuated using the vacuum pump.

### 2.3. Data Preprocessing

A total of 64 acquisitions (baseline, exposure, and recovery phase) of three surface materials (undoped ZnO, Mn-doped ZnO, and F-doped ZnO with 8, 36, and 20 acquisitions, respectively) exposed to two gases (ethanol and trimethylamine, 34 and 30 acquisitions, respectively) with three different ppm intervals (<20 ppm, 20–50 ppm and >50 ppm, 24, 16 and 24 acquisitions respectively) are obtained. Figure 1 shows the raw un-normalized sensor responses. The signal amplitude and response time vary greatly, e.g., the response from undoped ZnO reaches a maximum value after 6–14 min, F-doped ZnO reaches a maximum value after 1–5 min, and Mn-doped ZnO reaches a maximum value after just a few seconds. Table 1 shows a summary of all 64 acquisitions.

Preprocessing is done by subtracting each acquisition with the baseline value and dividing with the maximum value of all acquisitions for that material.

### 2.4. Dimension Reduction and Classification

**Figure 2.**
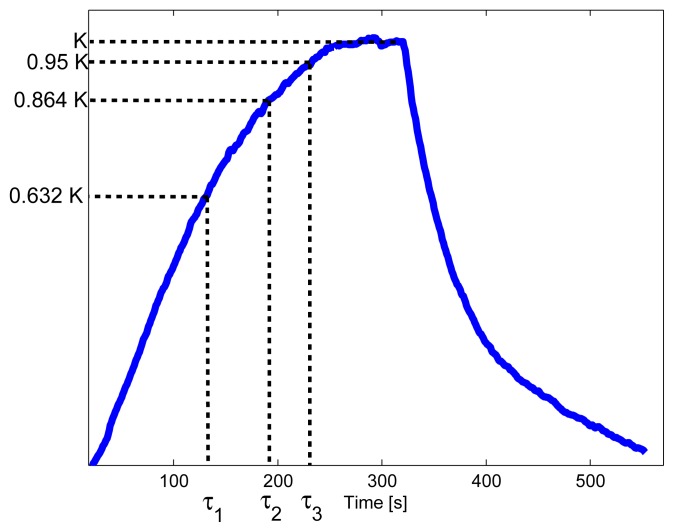
Depiction of feature extraction from one acquisition. See text for details.

For each acquisition, a set of features are extracted and used with three individually trained support vector machines (SVM) to classify each acquisition into material, gas and ppm level. A total of 7 features [38] are used: the maximum response (K), the first, second, and third time constant (*τ*_1_, *τ*_2_, and *τ*_3_ respectively), and the area under the response between 0 and *τ*_1_, *τ*_1_ and *τ*_2_ and *τ*_2_ to *τ*_3_, see Figure 2. Normalization of each feature is done by subtracting the mean and dividing with the standard deviation. Note that these features are based on knowing the maximum response, K.

A SVM with a Gaussian kernel is used, and cross-validation is used for selecting the model parameters *C* and *γ*. Comparison with Naive Bayes and a softmax classifier revealed that the SVM gave the best classification accuracy of the three.

### 2.5. Unsupervised Feature Learning

Deep neural networks (stacked restricted Boltzmann machines and auto-encoders) are employed in this work primarily because they offer the advantage of being unsupervised. Features are learned instead of being hand-designed, which can be a challenging task for gas sensor responses. Another possible advantage is the usage of self-taught learning [39] or transfer learning [40], which is a framework for training the model using additional examples not necessarily drawn from the same distribution as the samples of the classification task. This is especially useful when there are few labeled examples.

#### 2.5.1. Stacked Restricted Boltzmann Machine

**Figure 3.**
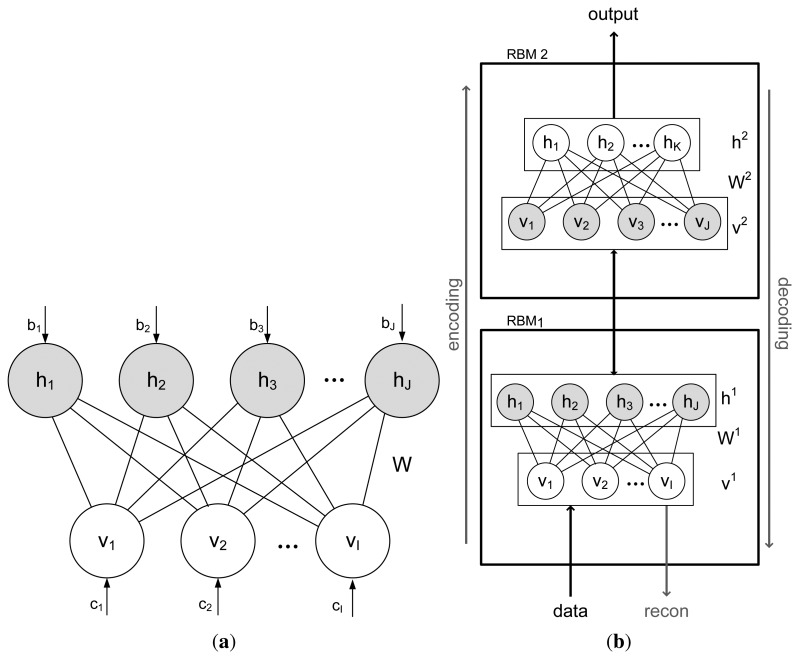
Graphical depiction of (**a**) RBM and (**b**) stacked RBM.

A Restricted Boltzmann Machine (RBM) is defined by restricting the interactions in the Boltzmann energy function [41] to only include connections between the input data and hidden units, *i.e.*, there are no visible-to-visible, or hidden-to-hidden connections, see Figure 3.

Modules of RBMs can be stacked on top of each other to form a deep belief network [42] (DBN). A DBN is a probabilistic undirected graphical model, where the model parameters are initialized by unsupervised greedy layer-wise training and the hidden layer from a lower-level RBM is the visible layer at the next level RBM. The layer of visible units (that represents the data), **v**, and hidden units, **h**, with corresponding bias vector, **c** and **b** are connected by a weight matrix, **W** and the energy function and joint distribution for a given visible and hidden vector are:

$$\begin{array}{r} E(\text{v},\text{h})={\text{h}}^{T}\text{Wv}+{\text{b}}^{T}\text{h}+{\text{c}}^{T}\text{v}\\ P(\text{v},\text{h})=\frac{1}{Z}\exp^{E(\text{v},\text{h})} \end{array}$$

where *Z* is the partition function that ensures that the distribution is normalized.

To feed our sensor data to a RBM we chose a window width, *w*, which will be the number of visible units, and let visible unit *υ_i_* represent data *x*(*t* + *i*) for *t* = 0…*T* − *w* where *x* is the data vector and *T* is the length of *x*.

For a Bernoulli–Bernoulli RBM (which assumes visible and hidden units in the range of [0, 1] and requires less data manipulation for our data and is simpler to implement and explain than a Gaussian–Bernoulli RBM), the feed-forward and feed-backward passes are given by:

$$\begin{array}{ll} P(h_{j}\;|\;\text{v}) & =\sigma \left(b_{j}+\sum_{i}W_{\text{ij}}\upsilon_{i}\right)\\ P(\upsilon_{i}\;|\;\text{h}) & =\sigma \left(c_{i}+\sum_{j}W_{\text{ij}}h_{j}\right) \end{array}$$

where *σ*(·) is the sigmoid activation function $\sigma (x)=\frac{1}{1+e^{-x}}$. The parameters **W**, **b**, and **v**, are trained using contrastive divergence [42], which is an approximation of the gradient of the log likelihood of **v**. The learning rule for RBM is

$$\frac{\partial \log P(\text{v})}{\partial W_{\text{ij}}}\approx {\langle \upsilon_{i}h_{j}\rangle }_{\text{data}}-{\langle \upsilon_{i}h_{j}\rangle }_{\text{recon}}$$

where 〈·〉 is the average value over all training samples.

After the initial pre-training of each layer, the network is finetuned with backpropagation. Both unsupervised, by minimizing the reconstruction error from the input to the top RBM and back, and supervised, by minimizing the classification accuracy on the training data. The learning objective in the fine-tuning step is heavily regularized in order to prevent overfitting and reduces the model complexity.

A Conditional Restricted Boltzmann Machine [43] (cRBM) is similar to a RBM except that the bias vectors for the visible and hidden layers is dynamic and depends on previous visible layers. While RBMs are used to learn representations of data, the cRBM can model temporal dependencies and is usually used for making predictions [44], in particular for multivariate data. The dynamic bias vectors are defined as:

$$\begin{array}{ll} b_{j}^{*} & =b_{j}+\sum_{i=1}^{n}B_{i}\upsilon (t-i)\\ c_{i}^{*} & =b_{j}+\sum_{i=1}^{n}A_{i}\upsilon (t-i) \end{array}$$

where *A_i_* is the autoregressive connections between visible layers at time *t* − *i* and current visible layer, *B_i_* is the weight matrix connecting visible layer at time *t* − *i* to the current hidden layer. The model order is defined by the constant *n*.

#### 2.5.2. Auto Encoder

An auto-encoder [45] consists of a layer of visible units, *υ*, a layer of hidden units, *h*, and a layer of reconstruction of the visible units, *υ̂*. The layers are connected via weight matrices *W*_1_ and *W*_2_ and the hidden and reconstructed layer have bias vectors *b*_1_ and *b*_2_, respectively. The hidden layer has a non-linear activation function, *σ*, in this case the sigmoid function $\sigma (x)=\frac{1}{1+e^{-x}}$. The reconstruction layer uses a linear activation function, *σ*(*x*) = *x*, which enables the visible layer to have values below zero and over 1. A feed-forward step in the network thus becomes

$$\begin{array}{ll} h & =\sigma (W_{1}\upsilon +b_{1})\\ \hat{\upsilon } & =W_{2}h+b_{2} \end{array}$$

The objective function for an auto-encoder is

$$J(\theta )=\frac{1}{2N}\sum_{i}{\left(\upsilon^{\left(i\right)}-{\hat{\upsilon }}^{\left(i\right)}\right)}^{2}+\frac{\lambda }{2}\sum_{i}\sum_{j}W_{ij}^{2}+\beta \sum_{j}\rho \log\frac{\rho }{p_{j}}+(1-\rho )\log\frac{1-\rho }{1-p_{j}}$$

where *p_j_* is the mean activation for unit *j* and *N* is the number of training examples. The first term is the square root error term, the second term is the weight decay term, and the third term is the sparsity penalty term [46]. Hyperparameters (*λ*, *β*, and *ρ*) are set by examining the model parameters, layer activations and classification accuracy on validation set from randomized hyperparameter initialization [47].

The cost function for supervised fine-tuning is the same as for unsupervised training except for the square error term which becomes

$$J_{1}=-\frac{1}{N}\sum \left(1-\hat{y})\log(1-y)+\hat{y}\log(y\right)$$

where *ŷ* is the training label vector.

The auto-encoder can be modified to resemble the structure of a cRBM in order to make it more suitable for multivariate time-series data. This is done by setting the new visible layer as the concatenation of current and previous visible layers. For the first layer, this is equivalent to using a window of data as input. For the second layer, however, this is equivalent to using a sequence of first hidden layers as input to the second layer. More precisely, with model order, *n_i_*, at layer *i* and signal data at time *t*, *s*(*t*), the visible layer in the first layer becomes

(1) $$\upsilon^{1}=\left[\begin{array}{cccc} s(n_{1}) & s(n_{1}+n_{1}) & \ldots & s(T_{1})\\ s(n_{1}-1) & s(n_{1}+n_{1}-1) & \ldots & s(T_{1}-1)\\ \vdots & \vdots & \ddots & \vdots \\ s(0) & s(n_{1}+1) & \ldots & s(T_{1}-n_{1}) \end{array}\right]$$

where *T*_1_ is the number of samples in the data.

For the second layer, the visible layer is the concatenation of previous hidden layers and becomes

(2) $$\upsilon^{2}=\left[\begin{array}{cccc} h^{1}(n_{2}) & h^{1}(n_{2}+n_{2}) & \ldots & h^{1}(T_{2})\\ h^{1}(n_{2}-1) & h^{1}(n_{2}+n_{2}-1) & \ldots & h^{1}(T_{2}-1)\\ \vdots & \vdots & \ddots & \vdots \\ h^{1}(1) & h^{1}(n_{2}+1) & \ldots & h^{1}(T_{2}-n_{2}) \end{array}\right]$$

where $T_{2}=\left\lfloor \frac{T_{1}}{n_{1}}\right\rfloor$ is the number of hidden layer 1 samples.

## 3. Results and Discussion

**Figure 4.**
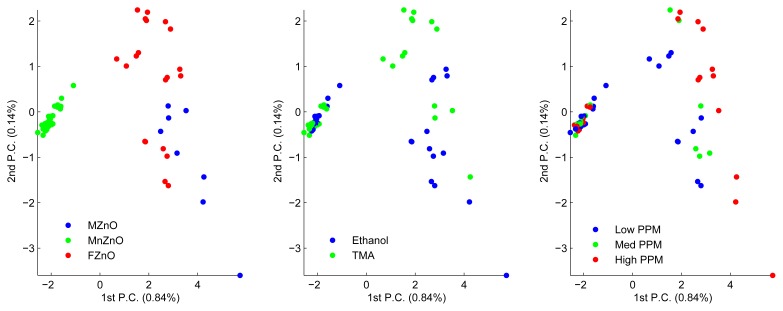
PCA of the data with 7 extracted features. Each point belongs to three categories, namely material of sensor, type of gas, and ppm level of the gas. For visualization purposes, three plots are shown where the data points have been colored according to category belonging.

**Figure 5.**
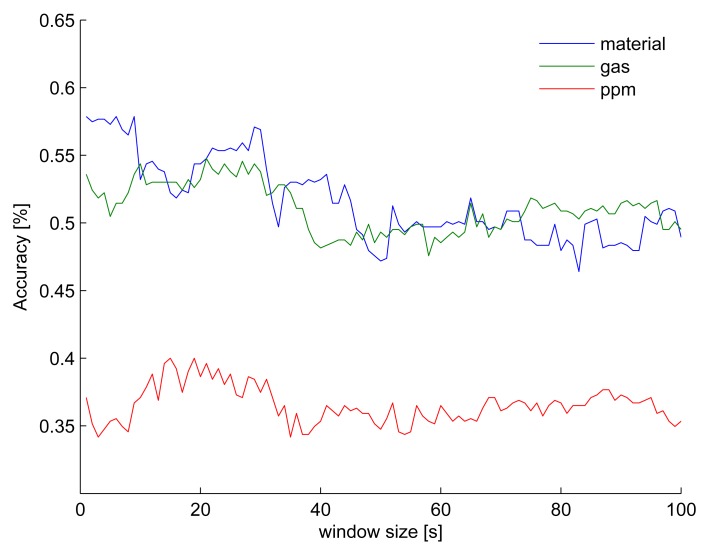
Classification accuracy when training a SVM on raw data with variable window size. The optimal window size is around 25 s.

Figure 4 shows three principal component analysis (PCA) with the same data but colored according to the three classification tasks: material, gas, and ppm level. These plots indicate that material is the easiest category to classify, followed by gas and, finally, ppm level. Figure 5 shows the classification accuracy when a support vector machine (SVM) is trained on (normalized) raw data with increasing window size. Using around 25 s of raw data with a SVM gives the best accuracy for the most difficult classification task. Therefore, a deep belief network (DBN) and auto-encoder are initially trained on a window of 25 s of input data and then incrementally decreased.

Table 2 shows the classification accuracies for a number of experiments. The accuracy with a SVM with seven features reaches 89.0%, 60.1% and 42.9% for the task of classifying material, gas and ppm level, respectively. However, this method requires the knowledge of the maximum response to extract the required features, which for some materials could take up to 14 min.

When using a two-layer unsupervised pre-trained and supervised fine-tuned DBN with the first 25 s of the response, the classification accuracy is 86.8%, 83.7% and 49.5%, which is better at classifying gas and ppm level and slightly worse at classifying the material compared to using a SVM with seven features. When the input window decreased, the classification accuracy decreases, as well. Before applying the DBN the data in the first 25 s where normalized in order to keep the values between 0 and 1. Additional training examples were obtained by shifting existing training examples.

A two-layer unsupervised pre-trained and supervised fine-tuned auto-encoder achieved 93.2%, 84.3%, 61.2% with the first 25 s of the response, which is already better than the feature-based approach on all three categories. The model uses a model order of 5 in both the first and second layer. With 10 s of input data (model order 5 in first layer and model order 2 in second layer) the accuracy becomes 95.7%, 80.8%, 51.1%, which is still higher than the SVM with seven features. Further decreasing the model order of the first layer reveals that a model order of 3 is better than the feature based approach but not a model order of 2. This means that the fastest classification at run-time, while still having comparable result with an approach where the maximum value is known, is 6 s of input data. When comparing the auto-encoder with a DBN, we see that given 25 s of input data to both models, the auto-encoder performs much better on all three tasks. Even when lowering the model order of the auto-encoder to 3 for first layer and 2 for second layer, resulting in using 6 s of data, the auto-encoder outperforms a DBN that uses 25 s of data. The main reason for this result is because our auto-encoder has been modified to better resemble a conditional RBM, which is more suitable for multi-variate time-series data than a standard DBN.

## 4. Conclusion

In this work, two deep network models, namely the DBN and an auto-encoder with concatenated previous visible layers, have been trained on multi-labeled data. The results have been compared to a feature-based approach where the maximum response is known. It was shown that the auto-encoder can achieve comparable results as the feature-based approach using only 6 s of input data. The DBN also achieves comparable results to the feature-based approach, though it requires more input data compared to the auto-encoder. In sum, this paper has shown that a deep network is able to give better and faster classification. Further, additional benefits arise when training a deep network. In the deep network, the unsupervised greedy layer-wise pre-training and unsupervised fine-tuning of the whole network is only performed once. When it is time to specialize the model to a specific task, the supervised fine-tuning step is performed for each classification task. In a SVM, on the other hand, the training has to be restarted for every classification task. Therefore, an unsupervised feature learning step is efficient for this kind of multi-label classification task.

Clearly for electronic nose applications, the selection of the materials used in the array is important to achieve good discrimination for the intended application. However, solving the problems related to the selectivity in gas-sensing applications still remains a major challenge. In this context, the different sensing behavior of F and Mn-doped ZnO thin films towards ethanol and TMA is encouraging and will help to solve the problem of selectivity. Though F/Mn-doped films are n-type semiconductors and ethanol/TMA are reducing gases, the presence of grain boundary scattering during sensing in the first case, and the absence of the same in the second case, helped to develop sensing elements with inherent selective nature. Adding such sensing elements to the array of sensors present in an electronic nose will provide new possibilities for detection of specific markers. Further, using these materials together with deep network models has shown to provide a classification performance (in accuracy and time) that is suitable for real-world deployment and comparable to current instruments on the market today.

## Figures and Tables

**Table 1.** Summary of all 64 acquisitions.

Material	Gas	ppm level	# acquisitions
ZnO	ethanol	low medium high	1 1 2
	TMA	low medium high	1 1 2
MnZnO	ethanol	low medium high	8 6 6
	TMA	low medium high	6 4 6
FZnO	ethanol	low medium high	4 2 4
	TMA	low medium high	4 2 4

**Table 2.** Classification accuracy (mean ± standard deviation) [%] with five-fold cross-validation for the task of classifying material, gas, and ppm level using different set-ups. The number after DBN defines the window width (number of visible units) and the numbers after auto-encoder define the model order in the first and second layer.

	Material	Gas	ppm	Required input data
SVM, 7 features	89.0 ± 6.3	60.1 ± 11.1	42.9 ± 7.6	15 s to 14 min
SVM, 25 s raw data	55.5 ± 3.7	53.8 ± 7.3	38.8 ± 4.1	25 s
DBN, 25 s	86.8 ± 4.6	83.7 ± 4.1	49.5 ± 5.6	25 s
DBN, 10 s	83.7 ± 1.9	61.4 ± 4.7	41.7 ± 6.8	10 s
DBN, 5 s	72.4 ± 3.5	60.0 ± 4.5	33.0 ± 3.3	5 s
Auto-encoder, 5-5	93.2 ± 3.6	84.3 ± 3.8	61.2 ± 6.8	25 s
Auto-encoder, 5-2	95.7 ± 1.3	80.8 ± 6.6	51.1 ± 1.6	10 s
Auto-encoder, 3-2	91.8 ± 3.2	71.1 ± 5.8	45.3 ± 4.5	6 s
Auto-encoder, 2-2	61.7 ± 1.5	45.4 ± 4.7	36.5 ± 3.5	4 s
